# Regression of portal hypertension: underlying mechanisms and therapeutic strategies

**DOI:** 10.1007/s12072-021-10135-4

**Published:** 2021-02-05

**Authors:** Sonia Selicean, Cong Wang, Sergi Guixé-Muntet, Horia Stefanescu, Norifumi Kawada, Jordi Gracia-Sancho

**Affiliations:** 1 Hepatology, Department of Biomedical Research, University of Bern, Inselspital, Murtenstrasse 35, Maurice E. Müller-Haus, F821a, 3008 Bern, Switzerland; 2 Department of Hepatology, Prof. Dr. Octavian Fodor Regional Institute of Gastroenterology and Hepatology, Liver Research Club, Cluj-Napoca, Romania; 3 Department of Hepatology, Graduate School of Medicine, Osaka City University, Osaka, Japan; 4 Liver Vascular Biology Research Group, IDIBAPS Research Institute, CIBEREHD, Barcelona, Spain

**Keywords:** Chronic liver disease, Cirrhosis, Hepatic hemodynamic, Hepatic circulation, Portal pressure, Liver sinusoid, NAFLD, NASH, Biomarker

## Abstract

Portal hypertension is the main non-neoplastic complication of chronic liver disease, being the cause of important life-threatening events including the development of ascites or variceal bleeding. The primary factor in the development of portal hypertension is a pathological increase in the intrahepatic vascular resistance, due to liver microcirculatory dysfunction, which is subsequently aggravated by extra-hepatic vascular disturbances including elevation of portal blood inflow. Evidence from pre-clinical models of cirrhosis has demonstrated that portal hypertension and chronic liver disease can be reversible if the injurious etiological agent is removed and can be further promoted using pharmacological therapy. These important observations have been partially demonstrated in clinical studies. This paper aims at providing an updated review of the currently available data regarding spontaneous and drug-promoted regression of portal hypertension, paying special attention to the clinical evidence. It also considers pathophysiological caveats that highlight the need for caution in establishing a new dogma that human chronic liver disease and portal hypertension is reversible.

## Introduction

Approximately 2 million people die each year from complications of chronic liver disease (CLD) in spite of recent major progresses in this field [1]. CLD originates due to chronic injury, which induces excessive extracellular matrix (ECM) deposition and microvascular dysfunction that, over time, hinder intrahepatic circulation and induce portal hypertension (PH) [2].

**Fig. 1:**
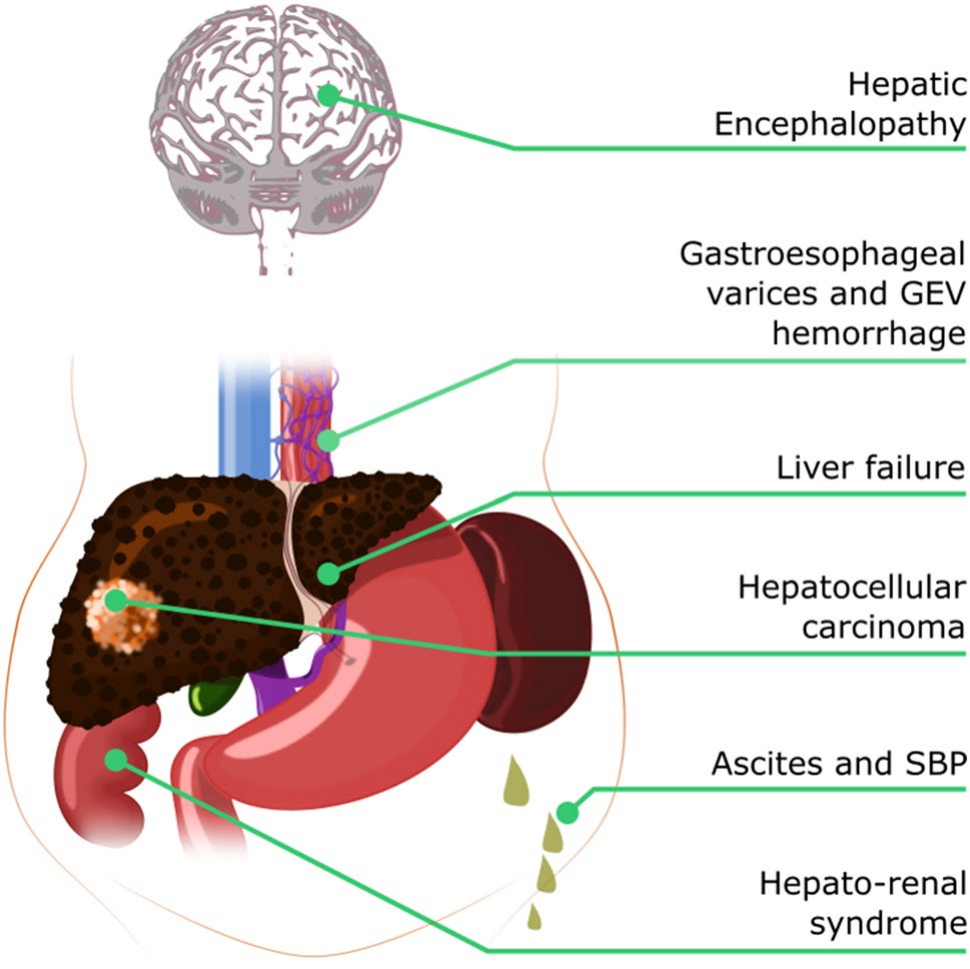
Schematic representation of complications of portal hypertension. Clinically significant portal hypertension may lead to hepatic encephalopathy, gastroesophageal varices prone to hemorrhage, liver failure, hepatorenal syndrome and ascites and it is also associated with an increased risk for hepatocellular carcinoma development. *GEV* gastroesophageal varices, *SBP* spontaneous bacterial peritonitis

PH is a clinical syndrome defined as an increased blood pressure in the portal venous system, being the primary cause of clinically relevant complications such as ascites, jaundice, variceal bleeding and an increased risk of spontaneous bacterial peritonitis or other bacterial infections, hepatic encephalopathy, hepatorenal syndrome and liver failure [3] (Fig. 1). The current gold standard for diagnosing and staging cirrhotic (sinusoidal) portal hypertension is HVPG measurement, which allows estimation of the portal pressure by calculating the difference between the wedged hepatic venous pressure (WHVP) and the free hepatic venous pressure (FHVP).

In normal conditions, HVPG ranges from 1 to < 5 mmHg. Values greater than 5 mmHg indicate portal hypertension; while, a HVPG greater than 10 mmHg indicates clinically significant portal hypertension (CSPH), which may result in the above-described life-threatening clinical complications [3].

**Fig. 2:**
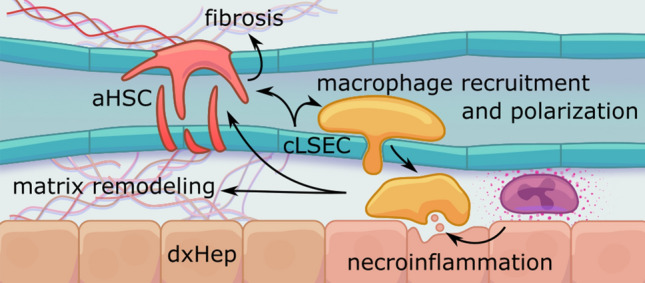
Main cellular mechanisms contributing to the progression of chronic liver disease in response to liver damage. Chronic liver injury induces the expression of cell adhesion molecules by LSEC, leading to a recruitment of macrophages to the tissue. These, together with liver damage, lead to necroinflammation, capillarization of LSEC, polarization of macrophages, HSC activation and liver fibrosis. *aHSC* activated hepatic stellate cell, *cLSEC* capillarized liver sinusoidal endothelial cell, *dxHep* dysfunctional hepatocyte

Initially, PH develops due to increased intrahepatic vascular resistance (HVR) to blood flow. This increased HVR is most commonly caused by chronic liver disease as a result of multiple pathological events in the sinusoidal circulation [4]. Indeed, during the process of liver injury, and regardless of the etiology, liver sinusoidal endothelial cells (LSECs) are rapidly de-regulated and begin to de-differentiate, acquiring a “capillarized” phenotype. They become proinflammatory and produce soluble factors that reach neighboring cells and determine their phenotype [5]. At the same time, exogenous liver injury induces a transcriptional change in hepatocytes, promoting their proliferation and death. This, in turn, leads to the release of apoptotic bodies that further contribute to the activation of other hepatic cells [6, 7]. Due to these injuring stimuli, hepatic stellate cells (HSCs) leave their quiescent state, becoming proliferative, pro-contractile and start synthesizing ECM components, becoming the most direct contributors to hepatic fibrosis. Persistent fibrosis, then, leads to distortion of the liver parenchyma and vascular structures, contributing to the stiffening of the organ and perturbing many cellular functions [8], ultimately leading to increased HVR and PH (Fig. 2). In advanced stages of the disease, the splanchnic tissue senses the reduced blood flow and increased pressure upstream of the liver, and produces an extrahepatic vasodilatory response as a compensatory mechanism. However, this leads to increased blood flow to the portal vein (hyperdynamic circulation), further aggravating PH [9].

Currently, therapeutics for PH consist of drugs targeting the extrahepatic vascular bed (mostly non-selective beta-blockers) that ameliorate systemic circulation and the hyperdynamic syndrome, leading to a reduction in portal blood flow. Despite the fact that these approaches may accomplish a reduction in PH in some cases, they do not have an effect on increased HVR (the primary cause of PH). Until recently, advanced fibrosis was thought to be irreversible, liver transplantation being the only option to cure CLD in most cases [10]. However, recent data from both human and animal models have challenged this view and suggest that after removal of the etiologic factor liver fibrosis and even cirrhosis may regress [11]. This is the case of a fraction of the patients cured from hepatitis B, hepatitis C, hepatitis Delta or metabolic hepatitis [12], that achieved normal or near-normal liver histology and function after the etiological agent was removed. Therefore, such insights may provide valuable data in determining the underlying mechanisms of PH and potential future therapeutic strategies.

## Regression of portal hypertension: spontaneous mechanisms

### Pre-clinical evidence for PH regression upon etiologic treatment

Spontaneous resolution of fibrosis has been observed in the gold-standard models of cirrhosis, including the carbon tetrachloride (CCl_4_), thioacetamide (TAA) and bile duct ligation models [13, 14]. Furthermore, regression of fibrosis and PH was also observed in animals with NASH after replacement of the high-fat diet for standard diet [15], altogether suggesting that reversion of fibrosis and PH may be achieved in different etiological backgrounds.

Indeed, Abdel-Aziz and colleagues studied the reversibility of fibrosis in experimentally induced cholestasis in rats. Three weeks after the ligation of bile ducts, they observed bile duct proliferation and periportal fibrosis. Three weeks after relief of the bile duct ligation, there was resorption normalization of periportal fibrosis, except for the persistence of collagen IV in the sinusoids [13]. Iredale and colleagues examined spontaneous recovery from liver fibrosis in CCl_4_-treated rats. After four weeks of induction and four additional weeks of recovery, they observed dissolution of the collagen fibrous matrix and a return to essentially normal liver structure [16]. Additional studies confirmed these observations [17, 18].

### Clinical evidence for PH regression upon etiologic treatment

As exposed above, one of the first and most important steps towards achieving regression of CLD is the removal of the injurious agent. The first observations regarding the issue date back in the early 1990s, when the resolution of esophageal varices was demonstrated in a small group of alcoholic cirrhotics who managed to abstain from alcohol for a long period of time [19]. Also, one-year alcohol abstinence determined a 46% reduction in hepatic vein wedge pressure (as a surrogate of portal pressure) [20]. The importance of achieving this goal has been demonstrated in CLD of several etiologies (Table 1), most convincingly in viral CLD.

**Fig. 3:**
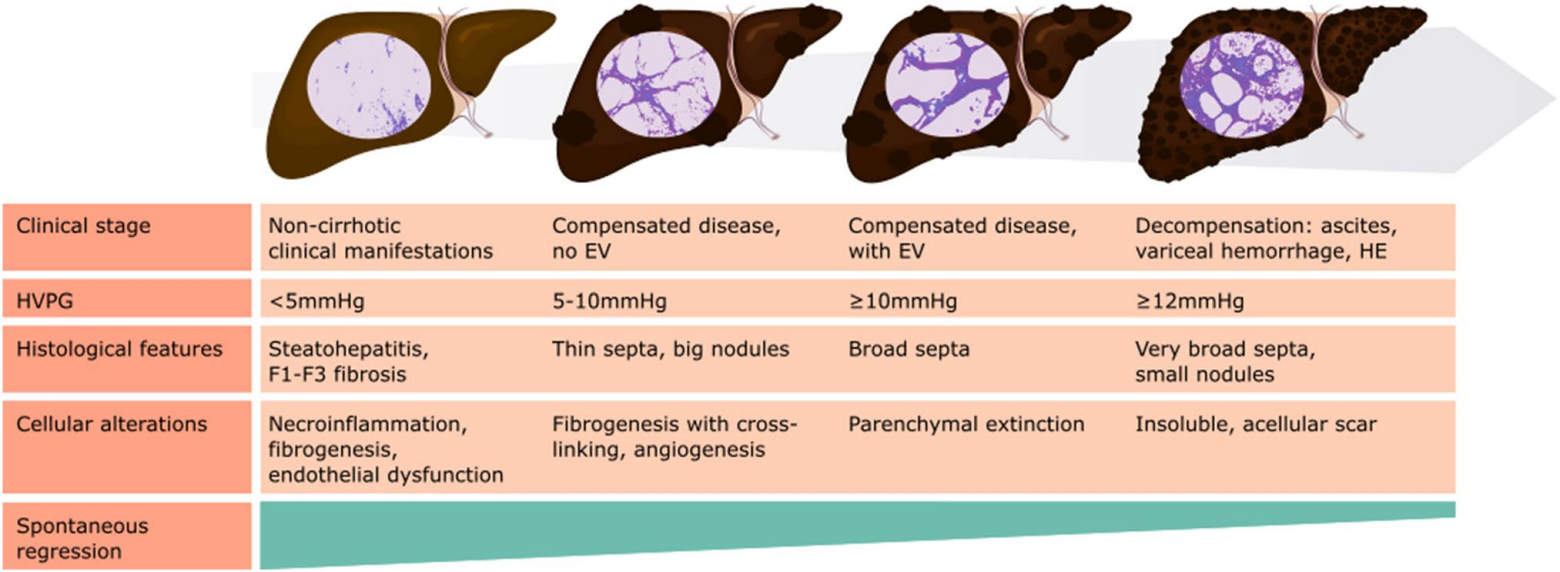
Stage-specific features determining the probability of regression of portal hypertension and chronic liver disease. It is accepted that the likeliness of regression is inversely correlated to the severity of the disease, usually determined by factors such as HVPG, thickness of fibrous septa or acellularization. *HVPG* hepatic venous pressure gradient, *HE* hepatic encephalopathy

- *HBV*: Long-term studies (5–7 years) showed a histologically proven progressive reduction in necroinflammation and fibrosis scores in a vast majority of antiviral treatment (AVT)-responsive patients with HBV advanced fibrosis or cirrhosis [21, 22]. In addition, several studies demonstrated the beneficial effects of viral suppression on non-malignant decompensating events [23, 24], low grade esophageal varices (EV) progression [25, 26], EV development rate [26], and clinical scores and transplant-free survival [27]. Although the evaluation of EV dynamics is based on subjective judgement, the evidence provided by these trials suggest an underlying decrease in PH. One study including 19 patients with HBV-related CSPH showed a median reduction of 18.7% in HVPG during the 12 months of follow-up, with no significant systemic hemodynamic changes [28], suggesting a reduction in HVR, possibly through decreased hepatic necroinflammation.
- *HCV, pre-DAA*: While sustained virological response (SVR) after HCV therapy is significantly higher since the introduction of direct-acting antivirals (DAA), several pre-DAA studies already held promise of PH regression induced by etiological intervention. As is the case with HBV AVT, treatment of HCV can also prevent the development of EV or slow down progression towards decompensation [29]. However, it seems that this effect is less consistent once EV is already present before treatment initiation, suggesting a reduced effect on already established PH [29, 30]. Moreover, in spite of PH decrease, decompensation is not always prevented and regression below the CSPH threshold is not always achievable, even with long-term follow-up (5.2 years after end of treatment) [31]. Short-term hemodynamic and/or histology studies in compensated patients demonstrated HVPG reduction paralleled by reduction in necroinflammatory scores, but either no or very weak reduction in fibrosis scores, possibly due to the short follow-up time [32, 33]. Around 60% of treated HCV patients with advanced fibrosis or cirrhosis followed up over a long time period (2–5 years) showed a progressive reduction in fibrosis score on repeated biopsy and significant reduction in scar collagen content, even in those without obvious fibrosis regression [34–36]. However, no changes in sinusoidal capillarization, as assessed by CD34 positivity or α-SMA staining, were observed, pointing towards lack of intrahepatic vascular remodeling [34].
- *HCV, DAA*: With the emergence of DAAs, even patients in advanced stages of CLD can achieve viral eradication, which leaves open the question whether and to what degree their disease can be reversed. It is now clear that SVR after DAA can result in clinically significant (≥ 10–20%) decrease in HVPG [37, 38], even in those patients with baseline CSPH and in difficult to treat populations, such as HCV-HIV co-infected patients [38]. However, this effect seems less pronounced and more heterogeneous in those patients with advanced-stage CLD (high Child–Pugh score, HVPG > 16 mmHg) [37, 39]. A study on post-transplant graft HCV reinfection showed fibrosis regression and HVPG decrease in 67% and 66% of patients which achieved SVR, however, among F4 biopsies, none of the patients displaying thick fibrous septa (Laennec C cirrhosis) had cirrhosis regression [40]]. Histological data show that HVPG reduction is lower in those patients who still have necroinflammatory activity after SVR [38], which points towards an initial phase of reduction in HVPG through decreased intrahepatic inflammation [41]. However, it seems that HVPG decreases progressively over a longer time period, suggesting a long-term possibility of further decrease in HVPG based on mechanisms other than reduced inflammation. Still, in a relatively high proportion of patients, CSPH did not resolve, leaving them a risk for future decompensation [42–44].
  A significant decrease in HVPG in patients without baseline CSPH is particularly important given its potential to prevent progression of disease (Fig. 3). Indeed, achieving SVR seems to decrease disease severity (Child–Pugh and MELD scores), decompensation risk and EV grade [39, 45, 46]. These important improvements in HCV-induced CLD management are also mirrored in the shift in transplant indication from the pre-DAA era and even within the last few years [47]. The effect of DAA on systemic hemodynamics is still not well established, with some studies showing mild but significant increase in MAP and systemic vascular resistance [37]; whereas, others described no influence [38].
- *NAFLD*: The vast majority of patients benefit from different weight loss surgery approaches, with normalization or reduction in tissue fibrosis [48, 49], although this is more evident in early fibrosis [50]. Lifestyle interventions have an important role in obese CLD patients (any etiology), with 42% of patients showing a significant decrease (≥ 10%) in HVPG from baseline after 16 weeks, paralleled by a decrease in insulin resistance and plasma leptin levels, giving an insight into possible mechanisms of regression in this patient population [51]. Moreover, this last study showed no change in portal blood flow, thus reinforcing the probable effect on HVR of etiological treatment in CLD. A subsequent study investigating the effects of physical activity in cirrhotic patients with CSPH (compensated or decompensated) also demonstrated a reduction in HPVG [52].

**Table 1:** Summary of studies investigating HVPG response to etiologic intervention

Etiology	Treatment/intervention	Study population	HVPG decrease	FU time	Observations
HBV [28]	Lamivudine	*n* = 19, all with BL CSPH	18.7%^a^	12 months	60% of patients had a clinically significant reduction in HVPG (≥ 20% from BL)
HCV [32]	Ribavirin + PegIFN	*n* = 20, 55% with BL CSPH (defined as 12 mmHg in this study)	28%^b^	24–48 weeks	All patients had a reduction of ≥ 10% from BL HVPG
HCV [31]	Ribavirin + PegIFN	*n* = 100, 74% with BL CSPH	7.7%^a^	5.2 years (median time)	40% of patients had a clinically significant reduction in HVPG (≥ 10% from BL) but 2/3 of patients still displayed CSPH, leaving them at risk of developing decompensation
HCV [39]	DAA (different regimens)	*n* = 104 (*n* = 60 with FU HVPG), 68% with BL CSPH	23%^b^	12–24 weeks	Normalization of HVPG in 63% (BL HVPG = 6–9 mmHg) and 43% (BL HVPG = 10–15 mmHg) of patients; 60% of patients with BL HVPG ≥ 16 mmHg did not resolve CSPH
HCV [37]	DAA (different regimens)	*n* = 226, all with BL CSPH	13%^a^	24 weeks	78% of patients did not reach CSPH resolution, remaining at risk for decompensation
HCV + HIV [38]	DAA (different regimens)	*n* = 22, 50% with BL CSPH	32%^b^	12 weeks	77% of patients had a clinically significant reduction in HVPG (≥ 10% from BL). HVPG response correlated with decrease in necroinflammatory activity
HCV [40]	IFN-based or IFN-free regimens	*n* = 112 patients with graft HCV reinfection after LT, 31% with BL CSPH	− 2.5 mmHg	12 months	Low probability of regression in those with BL CSPH (18%) and Laennec C histology (0%)
HCV [44] *this is a follow-up study of the patients in [37]	DAA (different regimens)	*n* = 117, only patients with CSPH at SVR	24.4%	96 weeks	53% of patients remained with CSPH
HCV [43] *this study partially follows up patients in [39]	DAA (different regimens)	*n* = 67, 74% with BL CSPH	20%^a^	8.8 months (median)	*n* = 19 patients also included in [39] underwent a 3^rd^ HVPG measurement, with an increase in the proportion of patients with resolved CSPH from 15 to 46%
Any etiology + obesity [51]	Nutritional intervention + moderate physical activity	*n* = 50, 72% with BL CSPH	− 1.6 mmHg	16 weeks	42% of patients achieved a reduction of ≥ 10% from BL HVPG
Any etiology [52]	Nutritional intervention ± physical activity	*n* = 22	− 2.5 mmHg	14 weeks	

Clinically significant reduction in HVPG defined as reduction of 10–20% from baseline, depending on study

FU follow-up; *DAA* direct acting antivirals, *BL* baseline, *CSPH* clinically significant portal hypertension, *LT* liver transplant

^a^Median % reduction in HVPG

^b^Mean % reduction in HVPG

### Cellular and molecular events limiting spontaneous regression of PH

**Fig. 4:**
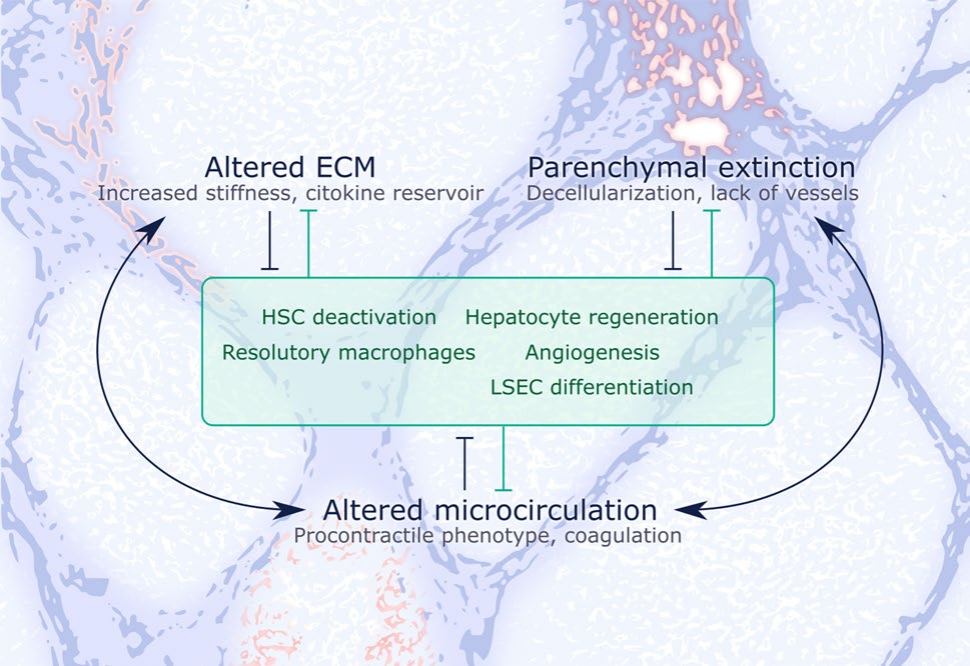
Molecular mechanisms modulating regression of chronic liver disease. Regression of cirrhosis and portal hypertension is usually impaired by liver architecture (altered extracellular matrix and acellularization) and microcirculatory dysfunction. All these may induce an activation response to hepatic cells, promoting progression rather than regression of chronic liver disease. On the other hand, modulation of the phenotype of hepatic cells (green box) may inhibit said alterations and represent potential targets for regression of chronic liver disease. *ECM* extracellular matrix, *HSC* hepatic stellate cell, *LSEC* liver sinusoidal endothelial cell

It is now evident that cirrhosis is at least partly reversible. However, the extent to which resolution can occur seems to be highly dependent on disease stage. Some of the most important elements that render PH resistant to regression are fibrotic tissue composition and stiffness, presence or absence of specific cell populations, and the profound (micro) vascular changes (Fig. 4), all elements which are significantly different in early versus advanced CLD.

- *Fibrotic tissue properties*: In a murine CCl_4_ model of CLD, followed up for 1 year after cessation of toxicant administration, regression was only partial, with transformation from micronodular to macronodular pattern and incomplete resolution of broad mature septa [14]. This mirrors changes described during human cirrhosis regression [53]. In humans, small nodule size and increased septal thickness have both been correlated with higher HVPG and seem to be predictive of decompensation [54] and patients with Laennec C cirrhosis are unlikely to have a significant HVPG decrease even after removal of etiological agent [40]. In murine models, enzymes such as tissue-transglutaminase (tTG) and lysil oxidases (LOX) induce the cross-linking of collagens and elastins, creating acellular areas that are resistant to degradation [14, 55, 56]. A study using tTG2 KO mice showed that this molecule is not indispensable during fibrogenesis and mice lacking it do not have a resolution advantage compared to wild-type mice [57]. This could suggest alternative collagen cross-linking pathways. In this regard, elastin also plays an important role in the irreversibility of advanced fibrosis. Elastin to collagen ratio increases with disease progression and, despite an early increase in expression, its marked accumulation only occurs in advanced stages. This points towards an imbalance between synthesis and removal, also suggested by the increase in matrix metalloproteinase 12 (MMP12) bound to tissue inhibitor of matrix metalloproteinase-1 (TIMP-1), which renders it inactive. Moreover, MMP12 KO mice display significantly higher level of bridging fibrosis, further suggesting a defect in elastin removal [58]. Depletion of macrophages results in additional accumulation of elastin compared to wild-type mice and with failure of tissue remodeling. Indeed, pro-resolution macrophages are an important source of MMPs [58, 59].
- *Role of HSCs*: Activated HSCs (aHSC) play a key role in fibrosis, and an important event necessary for its resolution is their disappearance through either senescence, apoptosis or inactivation [60]. HSCs situated within mature insoluble septa seem to be less prone to undergo apoptosis [14]. Indeed, it seems that persistence of scar tissue is associated with the maintenance of aHSC [61], which importantly also highlights the role of the underlying matrix in influencing cell phenotype [62]. aHSC are a major source of TIMPs and TIMP-1 overexpressing murine models fail to show resolution of fibrosis [63]. Moreover, it has been shown that TIMP-1 itself promotes survival of aHSC [64]. Although HSC inactivation may occur during fibrosis regression, these cells seem to remain more sensitive to renewed exposure to fibrogenic stimuli compared to their normal counterparts [65].
- *Vascular phenotype*: In addition to fibrosis, the other crucial component and the major causative factor of pathophysiological consequences of cirrhosis are the vascular changes, both intra- and extrahepatic. Hepatic endothelial de-differentiation and neo-angiogenesis depend on the initial injury pattern and it may well be that the endothelial and vascular changes are in fact the most important determinants of regression capacity [5, 66, 67]. As recently proposed by Wanless, advanced stage CLD can progress independent of the initial etiological agent, due to a vicious circle in which vascular injury promotes vascular obstruction which leads to renewed vascular injury and hepatocellular damage; the so-called ‘congestive escalator’ [68]. In line with this, a recent study in murine cirrhosis shows persistent liver hyperarterialization, in spite of cirrhosis regression [69].
- *Aging and other factors*: Advanced age has been shown to be an important determinant of CLD severity in murine models, results that were corroborated by HVPG and gene expression differences in human CLD patients with more advanced age [70]. Age seems to also be involved in CLD regression capacity, as shown in a murine CCl_4_ model, in which old mice were significantly less prone towards CLD reversal, as assessed by liver histology and ECM remodeling pathways (including macrophage populations) compared to their young counterparts [71]. Moreover, genetic and epigenetic factors likely play a role in the capacity and speed of CLD regression [72]. Last but not least, it is important to consider additional pro-fibrogenic factors which could influence the rate of regression in patients even after causative treatment, such as metabolic risk factors or excessive alcohol intake.

## Regression of portal hypertension: therapy-driven strategies

Despite the evidence of spontaneous regression after removal of the etiologic cause, there is still a great percentage of patients whose disease does not regress or even progresses. Therefore, in the recent years, there has been an increasing effort to develop new therapies that could have an impact in regression of cirrhosis and PH [73]. Indeed, regression of PH has been assessed by targeting the different intrahepatic alterations associated with the disease, which are the primary cause of increased HVR (Table 2). These approaches mainly achieve vasodilation or reduced inflammation, leading to amelioration of the dynamic and structural components of HVR.

**Table 2:** Effects of CLD therapies on portal hypertension regression

	Drug/substance	Drug/substance class	Effect on HVPG	Other effects
Beneficial effect on HVPG	Statins	Simvastatin [74–77, 79]	**↓, ↘**	↓ mortality, ↑ IGC
	Renin–angiotensin–aldosterone system antagonists	ARBs/ACEIs/AAs [82–84]	**↘**	Renal effects, ↓ MAP (CPS B and C), reduction in fibrosis progression
	Galectin 3 inhibitor	Belapectin [98]	**↘** (study without BL EV)	Prevention of de novo EV, ↓ hepatocyte ballooning
	FXR agonist	Obeticholic acid [100]	**↘**	
	Rho-kinase inhibitor	Fasudil [89]	**↘**	↓ SVR, ↓ MAP
	Multikinase inhibitor	Sorafenib [101]	**↘**	↓ VEGF, PDGF, PlGF, RhoA and TNFα mRNA levels
	Probiotic	VSL#3 [103–105]	≈/**↘**	↑ serum Na^2 +^ , ↓ plasma TNFα levels ↑ NSBB response rate
	Essential amino acid	Taurine [102]	**↘**	
	PDE-5 inhibitors	Udenafil [86]	**↓**	
		Vardenafil [85]	**↓**	
		Sildenafil [87, 88]	≈ (↓ HVR and ↑PBF)	↓ MAP
	Antioxidants	Dark chocolate [106]	**↓** Attenuation of postprandial HVPG increase	↑ MAP
		Ascorbic acid [107]	**↓** Attenuation of postprandial HVPG increase	
	Endothelin receptor antagonists	BQ-123 (ETA)—intrahepatic administration [94]	**↓**	
		Ambrisentan (ETA) [94]	**↓**	↓ MAP
No effect on HVPG	Endothelin receptor antagonists	Tezosentan [91] (dual ETA & ETB)	≈	
		BQ-123 (ETA) [92]	≈	↓ MAP, ↓ SVR
		BQ-788 (ETB) [92]	≈	↑ MAP, ↑ SVR
	LOXL2 inhibitor	Simtuzumab [108–110]	≈	
	Pan-caspase inhibitor	Emricasan [112, 113]	≈	
	Tetrahydrobiopterin analog	Sapropterin [96]	≈	
	Relaxin-2 analog	Serelaxin [97]	≈ (trial stopped prematurely)	
	Antibiotics	Norfloxacin [114–116, 121]	≈	↑ MAP, ↑ SVR
		Rifaximin [117–120]	Undetermined	↓ inflammation and bacterial translocation serum markers additive effect to b-blocker therapy

Treatment strategies are classified according to their effect on HVPG (beneficial effect—acute/chronic hemodynamic response—or no effect) and the number/size of the existing studies; **↓** = acute hemodynamic response; **↘** = chronic hemodynamic response; **≈** = no effect on HVPG; *MAP* mean arterial pressure, *SVR* systemic vascular resistance, *HVR* hepatic vascular resistance, *PBF* portal blood flow, *IGC* indocyanine green clearance, *EV* esophageal varices, *BL* baseline

### Vasomodulators

*Statins*: the first studies testing statins in humans evaluated acute hemodynamic responses to simvastatin and demonstrated a decrease in estimated HVR accompanied by increased hepatic NO production and a 50% reduction in post-prandial splanchnic hyperemia [74]. Longer periods of simvastatin treatment contributed to a significant decrease in PP as measured by HVPG [75, 76], to an improvement in liver function measured as IGC [75] and to a significant survival benefit related to decreased mortality due to EV bleeding and infection [77]. Importantly, the decrease in HVPG was further augmented when statins were used in combination with propranolol. Similarly, a study investigating simvastatin addition to carvedilol non-responders had positive results, increasing the number of patients with hemodynamic response [78]. In contrast, a recent study in the same patient category, using carvedilol in combination with simvastatin over a period of 3 months failed to show any additional benefit of combination therapy in reducing HVPG [79]. Ongoing trials are further testing statins in the context of CLD [80, 81].

*Renin–angiotensin–aldosterone system (RAAS) inhibitors*: angiotensin converting enzyme inhibitors (ACEIs), angiotensin receptor blockers (ARBs) and aldosterone antagonists (AAs) have shown beneficial effects on fibrosis in other organs. In CLD, they could potentially target the excess sodium and water retention while acting as antifibrotics. NAFLD patients under treatment with ACEIs or ARBs had slower fibrosis progression rate, more pronounced in a subgroup with concomitant type 2 diabetes mellitus [82]. Conversely, in the HALT-C cohort, a post hoc analysis did not show any differences in fibrosis progression compared to untreated controls [83]. Regarding their effect on HVPG, it seems that ARBs and/or ACEIs (possibly with the addition of mineralocorticoid antagonists) could be useful in Child–Pugh A patients, but probably not in more advanced, where the risk of adverse events (renal, electrolytic disturbances, hypotension) is much greater [84].

*PDE5 inhibitors*: Clinical results using several PDE5 inhibitors are contradictory, with some studies showing an acute HVPG decrease [85, 86], and other observing no change in HVPG probably due to simultaneous increase in PBF counteracting the decrease in HVR [87]. Considering certain systemic effects of PDE5 inhibitors [88], and that even a relatively minor decrease in MAP can be deleterious in patients with advanced CLD, the combination of these agents with NSBB may be unsafe in advanced CLD. This approach might be promising in patients in early disease stages, given the importance of HVR as pathogenetic mechanism and the still relatively normal systemic hemodynamic.

*Rho-kinase inhibitors*: Fasudil produced a statistically significant acute hemodynamic response in HVPG and reduction in portal vascular resistance in a small RCT, but with negative systemic hemodynamic effects evidenced by significantly decreasing MAP and SVR [89].

*Endothelin antagonism* has shown promising results in treating PH complications, such as hepatorenal syndrome and portopulmonary hypertension [90]. Regarding PP, continuous infusion of tezosentan (dual endothelin receptor antagonist) did not cause relevant changes in HVPG, hepatic blood flow or IGC in a cohort of cirrhotic patients with CSPH [91]. ETA or ETB receptor antagonists in Child–Pugh A cirrhotic patients showed opposing actions on systemic hemodynamics: while blocking of ETA decreased MAP, MPAP and systemic vascular resistance, inhibition of ETB increased MAP and SVR, with no effect on pulmonary hemodynamics [92], but no net effect on HVPG was observed. Since there might be a shift in the ETA to ETB receptor ratio in the liver during the development of cirrhosis [93], and the responsiveness to ET1 may be altered during the course of CLD, a more selective targeting of this pathway is required. Indeed, a recent study has shown a beneficial effect of intrahepatic administration of ETA antagonist, highlighting the important local effects. Additionally, the same study has demonstrated the efficacy of ambrisentan on lowering PP, without clinically significant systemic changes, even in advance CLD [94]. Currently, a clinical trial investigating the effect of the ETA antagonist ambrisentan on PH is recruiting patients [95].

*Sapropterin*, an oral synthetic analog of tetrahydrobiopterin (BH_4_), which is an essential co-factor for NO synthesis and is reduced in cirrhotic livers, has not shown an effect on HVPG, IGC or markers of endothelial dysfunction and oxidative stress during a 2-week period of treatment in cirrhotic patients with CSPH [96].

*Serelaxin*, a recombinant human relaxin-2 analog has demonstrated no effect in HVPG acute response; however, the trial cohort was small due to permanent drug discontinuation [97]. It is conceivable that, in an accurately powered trial, the drug might show an effect on PP.

### Therapies leading to reduced inflammation/fibrosis

*Belapectin*: this galectin 3 inhibitor did not show significant changes in HVPG, liver histology or development of complications in a phase 2 NASH trial, with the exception of a subgroup of patients without band-ligated varices, where there was a significant decrease in HVPG and in the development of de novo EV [98]. There is an ongoing trial in this category of patients [99].

The FXR agonist obeticholic acid (OCA) is currently under evaluation in several trials. In a small trial presented in abstract form, OCA treatment for 7 days significantly reduced HVPG in more than half of the cohort of alcoholic cirrhosis patients investigated, opening an avenue for future investigations in this direction [100].

*Sorafenib*, a multikinase inhibitor used as HCC therapy, decreased baseline HVPG by ≥ 20% in 36% of cirrhotic patients with HCC after 2 weeks of treatment. Moreover, it significantly decreased liver tissue mRNA levels of VEGF, PDGF, PlGF, RhoA kinase and TNFα [101].

*Taurine*: In a trial of 28-day taurine treatment in cirrhotic patients with CSPH, 58% of treated patients had a drop of ≥ 10% in HVPG, without any systemic hemodynamic effects [102].

*Probiotics*: While some authors described an important decrease in HVPG after VSL#3 probiotic treatment, with a higher proportion of patients achieving hemodynamic response in case of concomitant NSBB treatment [103], other patients seem to not benefit from this [104]. In addition, other beneficial effects of VSL#3 have been described, such as improvement in systemic hemodynamics, decrease in plasma TNFα levels [105] and improvement in serum Na^+^ concentration [103].

*Antioxidants*: acute administration of dark chocolate decreased the magnitude of postprandial increase in HVPG when compared to the control group, without significantly affecting PBF, suggesting a possible intrahepatic mechanism of action. Moreover, patients receiving dark chocolate had a mild increase in mean arterial pressure [106]. Similarly, a clinical trial using ascorbic acid attenuated post-prandial increase in HVPG without changing HBF [107].

*Simtuzumab*, a monoclonal antibody against lysis oxidase-like 2 (LOXL2), has shown no effect on any of the study endpoints in NASH fibrosis or cirrhosis, HCV/HIV or primary sclerosing cholangitis [108–110]. However, targeting LOX family members could still be a promising approach, as highlighted in a recent review [111], with new molecules engaging intracellular LOXL2 or targeting several other LOX family members.

*Emricasan*, a pan-caspase inhibitor, significantly decreased HVPG and aminotransferases in a subgroup of cirrhotic patients (any etiology) with CSPH ≥ 12 mmHg in an exploratory study [112]. However, in a follow-up RCT, emricasan did not achieve a significant reduction in HVPG in NASH cirrhosis patients with severe PH [113].

*Antibiotics*: 4 weeks of norfloxacin treatment was either non-superior to placebo or showed a non-significant trend towards HVPG reduction [114–116]. However, norfloxacin showed systemic hemodynamic effects in these studies, by increasing SVR and MAP. It is possible that, due to decreased NO caused by attenuated bacterial translocation, norfloxacin causes a decrease in portal blood flow and an increase in HVR, which counteract each other and could explain the lack of HVPG effect [114, 116]. Rifaximin has shown a significant effect on short-term HVPG decrease [117] and also reduced the risk of developing complications and improved survival when administered for up to 5 years [118]. However, this last study included only the HVPG responders from the previous short-term study, which might bias results. Conversely, a more recent RCT failed to demonstrate any effect of short-term rifaximin treatment on hepatic or systemic hemodynamics [119]. It seems that addition of rifaximin to NSBB has a further favorable effect [120].

### Studies not evaluating HVPG

Several studies, although not directly assessing HVPG reduction, have investigated the potential of different drugs to modify either components of CLD, such as fibrosis, or the natural history of the disease (Table 3).

**Table 3:** Studies evaluating regression of CLD but not evaluating portal hypertension

Drug (drug class)	Effects
Enoxaparin (anticoagulant) [122]	↓ probability of PVT development ↑ survival *A trial with rivaroxaban, another anticoagulant, is currently ongoing [123]
Liraglutide (GLP-1 analog) [124]	↓ progression of fibrosis (but no significant improvement) NASH resolution
Selonsertib (ASK1 inhibitor) [125]	No effect on fibrosis
Cenicriviroc (CCR2 and CCR5 antagonist) [126]	Improvement in fibrosis (effect more pronounced on patients with more advanced disease) ↓ in collagen area by morphometry, ↓ in systemic inflammation biomarkers *currently tested as monotherapy in a phase 3 trial (AURORA) or in combination with the FXR agonistc(TANDEM trial) in F2/3 NASH patients [127, 128]
Pioglitazone (PPAR γ agonist) and vitamin E [129]	Improvement in NASH (vitamin E but not pioglitazone) No improvement in fibrosis for any of the trial drugs
Lanifibranor (PPAR α/δ/γ agonist) [130]	NASH resolution Improvement in fibrosis
G-CSF or G-CSF followed by CD133 + cells (cell therapy) [131]	No improvement in liver function tests, non-invasive fibrosis markers, MELD or CPS

*PVT* portal vein thrombosis, *GLP-1* glucagon-like peptide-1, *ASK1* apoptosis signal-regulating kinase 1, *CCR* C-C chemokine receptor, *FXR* farnesoid X receptor, *PPAR* peroxisome proliferator-activated receptors, *G-CSF* granulocyte-colony stimulating factor, *CD* cluster of differentiation

## Biomarkers of PH regression

While HVPG measurement remains the gold-standard approach for monitoring the dynamics of PP, a multitude of non-invasive tests have been designed and evaluated for diagnosis, stratification of disease severity and progression monitoring [132]. However, these are likely inaccurate for evaluating regression of fibrosis and PH [72, 133]. Furthermore, treatment or removal of etiological agents can modify individual score components independently of the evolution of the underlying CLD [134, 135]. A summary of studies investigating noninvasive markers can be found in Table 4.

**Table 4:** Biomarkers of portal hypertension regression

Biomarker	Short description of study
Liver stiffness [39]	In a cohort of DAA-treated HCV patients, LS decrease (measured by TE) was associated with HVPG response. However, its accuracy was lower in patients with BL CSPH
Liver stiffness [37, 44]	In a cohort of DAA-treated patients, LS ≥ 21 kPa had a good performance in ruling in the persistence of CSPH after SVR (positive predictive value 82–91%); however, the lower cut-off of 13,6 kPa did not perform well in ruling out CSPH persistence
Liver stiffness [136]	In this small cohort of DAA-treated HCV patients, a cut-off value of < 12 kPa was accurate in ruling out CSPH after SVR
Liver stiffness and ELF score [40]	In a cohort of LT patients with HCV reinfection, LS was accurate in ruling in our out the persistence of CSPH (cut-off values < 11.3 and > 23 kPa resp) and the persistence of advanced fibrosis. Conversely, ELF showed good accuracy for CSPH, but was not associated with fibrosis regression
Liver stiffness and VITRO score [43]	In this cohort of DAA-treated HCV patients, TE and VITRO score performed well in ruling in/out CSPH after SVR. Their accuracy was especially high if used in a sequential manner, leaving 25% of patients unclassifiable
Spleen stiffness [137]	In this cohort of DAA-treated HCV patients, SS decreased significantly after SVR, more so in patients without BL CSPH. However, the presence and grade of PP was estimated based on LS, and no direct HVPG measurements were performed
Spleen stiffness [138]	This proof-of-concept study demonstrated that a decrease in spleen stiffness accurately predicts the hemodynamic response to primary prophylaxis with NSBB (Carvedilol) in patients with HREV. A prediction model containing SS had an AUC > 0.8 in both derivation and validation cohorts

*ELF score* Enhanced Liver Fibrosis score, which measures hyaluronic acid (HA), procollagen III amino-terminal peptide (PIIINP), and tissue inhibitor of matrix metalloproteinase 1 (TIMP-1), *HREV* high-risk esophageal varices, *LS* liver stiffness, *SS* spleen stiffness, *TE* transient elastography, *VITRO* von Willebrand antigen to platelet ratio score

Regarding the monitoring of CLD complications during PH regression, recently, the Baveno VI criteria for EV surveillance have been validated in HBV and/or HCV compensated patients, post-SVR [139].

Serum biomarkers of fibrogenesis and fibrolysis are another area of research. Collagen fragments can serve as such biomarkers: PRO-C3 and C6M have been shown to identify progressors, while PRO-C5 identified fibrosis regressors [140]. Moreover, combinations of these markers have been shown to correlate with the degree of portal hypertension [141, 142]. However, if and to what extent these markers correlate with HVPG reduction and clinical outcomes during cirrhosis regression, especially in patients with advanced CLD, remains a subject of future investigations.

## Conclusions and future perspective

Alcohol, NASH and viral hepatitis are the most common etiologies of CLD. Even if these are usually treatable (even more so after the recent development of direct antiviral strategies), removal of the etiologic agent may stop progression of the disease and lead to regression of fibrosis only in some of the cases.

The mechanisms of cirrhosis regression are still widely unknown, in part due to the limitations of preclinical models, which develop and partially revert cirrhosis in a short time as opposed to the slow clinical progression and regression of the disease. This is also true for the study of pharmacologic therapies, which are usually prophylactic during the pre-clinical induction of CLD or administered for a short time after mild disease is established, while in the case of clinical trials require consistent study design and usually repeated assessment with invasive techniques like liver biopsy. Other factors including the age and gender of the animals used in pre-clinical studies may also play a key role for future successful developments [143].

Despite these limitations, new advances in the study of PH regression point towards a clear role of the sinusoidal biomechanical properties in CLD, which could determine the cellular phenotype, vascular function, proliferation and overall drive the disease towards progression or regression.
